# The Association Between Chronic Tobacco Smoking and Brain Alterations in Schizophrenia: A Systematic Review of Magnetic Resonance Imaging Studies

**DOI:** 10.1093/schbul/sbae088

**Published:** 2024-06-02

**Authors:** Merel Koster, Lilli Mannsdörfer, Marieke van der Pluijm, Lieuwe de Haan, Tim Ziermans, Guido van Wingen, Jentien Vermeulen

**Affiliations:** Department of Psychiatry, Amsterdam UMC, University of Amsterdam, Amsterdam, The Netherlands; Department of Psychiatry, Amsterdam UMC, University of Amsterdam, Amsterdam, The Netherlands; Department of Psychiatry, Amsterdam UMC, University of Amsterdam, Amsterdam, The Netherlands; Department of Psychiatry, Amsterdam UMC, University of Amsterdam, Amsterdam, The Netherlands; Department of Psychology, University of Amsterdam, Amsterdam, The Netherlands; Department of Psychiatry, Amsterdam UMC, University of Amsterdam, Amsterdam, The Netherlands; Department of Psychiatry, Amsterdam UMC, University of Amsterdam, Amsterdam, The Netherlands

**Keywords:** schizophrenia spectrum disorders, magnetic resonance imaging, tobacco smoking, brain structure, brain function

## Abstract

**Background and Hypothesis:**

The high co-occurrence of tobacco smoking in patients with schizophrenia spectrum disorders (SSD) poses a serious health concern, linked to increased mortality and worse clinical outcomes. The mechanisms underlying this co-occurrence are not fully understood.

**Study Design:**

Addressing the need for a comprehensive overview of the impact of tobacco use on SSD neurobiology, we conducted a systematic review of neuroimaging studies (including structural, functional, and neurochemical magnetic resonance imaging studies) that investigate the association between chronic tobacco smoking and brain alterations in patients with SSD.

**Study Results:**

Eight structural and fourteen functional studies were included. Structural studies show widespread independent and additive reductions in gray matter in relation to smoking and SSD. The majority of functional studies suggest that smoking might be associated with improvements in connectivity deficits linked to SSD. However, the limited number of and high amount of cross-sectional studies, and high between-studies sample overlap prevent a conclusive determination of the nature and extent of the impact of smoking on brain functioning in patients with SSD. Overall, functional results imply a distinct neurobiological mechanism for tobacco addiction in patients with SSD, possibly attributed to differences at the nicotinic acetylcholine receptor level.

**Conclusions:**

Our findings highlight the need for more longitudinal and exposure-dependent studies to differentiate between inherent neurobiological differences and the (long-term) effects of smoking in SSD, and to unravel the complex interaction between smoking and schizophrenia at various disease stages. This could inform more effective strategies addressing smoking susceptibility in SSD, potentially improving clinical outcomes.

## Introduction

The prevalence of tobacco smoking among patients with schizophrenia is nearly 70%, representing a 2–3 times higher rate than that in the general population.^1^ Compared with patients with other psychiatric conditions, smoking rates are highest among patients with schizophrenia,^1^ while smoking cessation rates are lowest.^2^ Smoking-related illnesses are the leading preventable cause of death in schizophrenia,^3^ and smoking is associated with worse clinical outcomes such as psychotic relapse and readmission.^4^ Moreover, smoking patients have higher levels of depressive,^5^ positive,^6^ and negative^7^ symptom severity, and lower quality of life^6^ compared with nonsmoking patients. As such, understanding the relationship between smoking and schizophrenia could improve patient’s overall health outcomes by targeting potential mechanisms underlying smoking vulnerability in schizophrenia.

The etiology underlying the schizophrenia-smoking co-occurrence is complex and not fully understood. Multiple non-mutually exclusive hypotheses have been proposed to explain the co-occurrence, eg, focusing on the influence of shared genetic and environmental vulnerability,^8^ misattributions of relief of anxiety and stress to nicotine use,^9^ and using nicotine to alleviate cognitive deficits or antipsychotic side effects.^10^ However, even before the onset of illness, individuals who later develop schizophrenia exhibit a higher prevalence of smoking than observed in the general public,^11^ as do nonpsychotic relatives of those with schizophrenia.^12^ As these findings were adjusted for education,^11^ or groups were matched based on education levels,^12^ they suggest that the increased prevalence of smoking in people with schizophrenia is not solely due to environmental factors or medication side effects. Instead, it is likely influenced by shared neurobiological and genetic vulnerability.^13^

Neuroimaging research investigating smoking in schizophrenia offers insights into the underlying common neurobiological mechanisms and consequences of smoking in patients, aiming to inform the development of pharmacological and health interventions to improve smoking cessation, clinical outcomes, and life expectancy of patients with schizophrenia. Furthermore, the high smoking rate in patients with schizophrenia raises questions about potential neurobiological alterations induced by chronic tobacco use and its impact on the course and manifestations of the disorder. Therefore, research has sought to investigate the neurobiology of this relationship noninvasively using different magnetic resonance imaging (MRI) modalities.

While reviews have explored MRI studies on the pathology of schizophrenia and the effects of smoking on the brain separately^14,15^ or limitedly,^16^ a comprehensive overview of the conjunction is missing. To address this gap and provide a more complete overview, we conducted a systematic review of neuroimaging studies that investigate the association between chronic tobacco smoking and brain alterations in patients with schizophrenia. We focus specifically on chronic tobacco exposure as we are interested in understanding the long-term effects of natural smoking behavior and its health implications, as opposed to the acute agonistic nicotine effects. By systematically collating and integrating results across MRI modalities, including structural, functional, and neurochemical MRI, we aim to create a comprehensive understanding of the potential neurobiological consequences of chronic tobacco smoking in schizophrenia. We expect that, due to the cumulative neurotoxic effects of prolonged tobacco exposure, chronic smoking has an increasing negative effect on the neurobiological abnormalities associated with schizophrenia. We outline methodological limitations and challenges in the field, offering suggestions for future efforts. Additionally, we carefully examined the studies’ methods, risk of bias, and funding sources. The latter is because the tobacco industry has funded both internal and external research,^17^ which warrants a cautious and critical review of the literature.

## Methods

This review was performed following the Preferred Reporting Items for Systematic Reviews and Meta-analyses (PRISMA) guidelines.^18^ The protocol was registered in the Prospective Register of Systematic Reviews (PROSPERO) database (registration number CRD42023457387).

### Search Strategy

PsycINFO, Web of Science, PubMed, and Biosis were searched from inception to the 7th of June 2023. See supplementary material S1 for the full search syntax and outcomes per database. Study selection was performed independently by 2 researchers (M.K. and L.M.) using Rayyan.^19^ All titles and abstracts of retrieved publications were screened by M.K. and L.M. to identify eligible studies. Full-text articles were obtained and discussed in consensus meetings in case of inconsistencies. Inclusion criteria were based on the PICO framework and as follows: (1) *Population* was patients diagnosed with a schizophrenia spectrum disorder (SSD; including schizophrenia, schizoaffective disorder, schizophreniform disorder, and psychotic disorder not otherwise specified), (2) *Interventions or exposure* were current habitual cigarette smoking established through self-report or standardized questionnaires, without acute nicotine administration prior or during the MRI scan, (3) *Comparison or control* group were smoking or nonsmoking controls or nonsmoking SSD patients, and (4) *Outcomes* were metrics of structural, functional, or neurochemical MRI. See supplementary material S2 for an elaboration on the different MRI modalities. Subsequently, studies were excluded during full-text reading if (1) the article was written in a language other than English, (2) the publication was a meta-analysis, review, conference abstract, or not available in full text, and (3) if the statistics were not reported separately for smoking and nonsmoking subjects. Furthermore, we included studies investigating acute nicotine effects in full-text screening to enable potential data extraction on the chronic effects of tobacco before nicotine administration. However, acute nicotine effects are beyond the scope of this review, and studies that did not provide data prior to administration were also excluded. Lastly, reference lists of selected articles were screened (forward and backward tracking of the literature up to February 2024) for potential additional studies.

### Data Extraction

Data were extracted by M.K. and L.M. using identically structured forms (see supplementary material S3 for the item list). Briefly, information was extracted on the study population (including number of subjects per group, age, gender, included diagnoses, medication information, details of tobacco use), study design (including MRI technique, regions of interest), MRI acquisition (including image sequence, task information), method of analysis, main findings, and funding. Besides differences between groups in MRI outcomes, we also explored differences between groups in clinical and cognitive outcomes. The extracted data were compared between the reviewers to ensure accuracy. The assessment of methodological aspects was conducted by considering the Guidelines for Rating the Quality of Evidence (GRADE)^20^ and following the recommendations provided by the Committee on Best Practice in Data Analysis and Sharing (COBIDAS).^21^

### Quality Assessment of Included Studies

The NIH Quality Assessment Tool *for Observational Cohort and Cross-Sectional Studies* was used to assess the risk of bias for each of the included studies (https://www.nhlbi.nih.gov/health-topics/study-quality-assessment-tools). Studies were quantified as poor, fair, or good based on the tool criteria.

### Analysis

A meta-analysis will be conducted for each MRI modality if at least 3 studies include data for independent samples and at least one of the same brain regions.

## Results

**Fig. 1.**
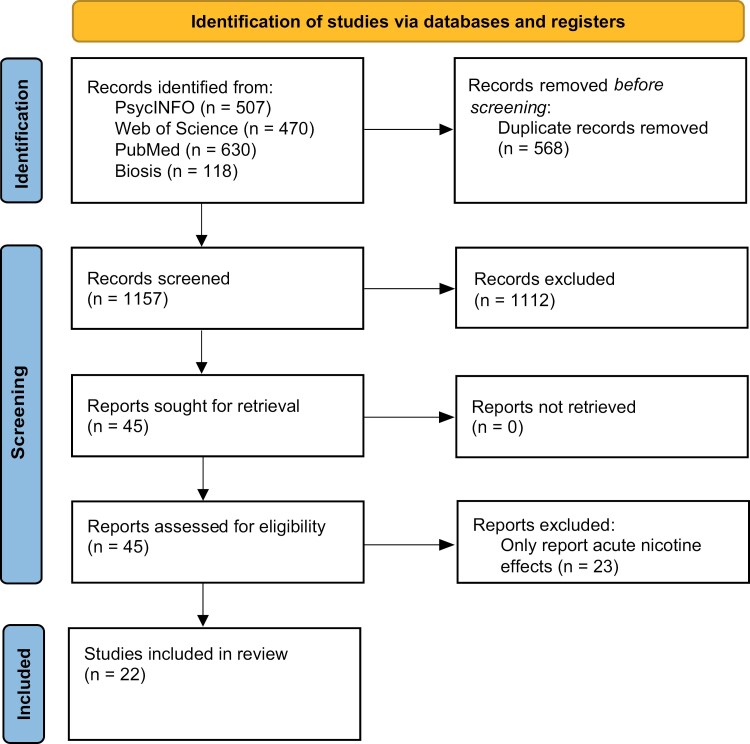
PRISMA flow diagram of study selection.

The selection of the articles is summarized in the PRISMA flow diagram (figure 1). Twenty-two studies met the abovementioned criteria, of which 8 structural imaging (including 2 diffusion tensor imaging [DTI] studies), 8 resting-state functional MRI (fMRI) studies, and 6 task-based fMRI. All studies had a naturalistic cross-sectional design except for 1 longitudinal structural imaging study.^22^ We found no studies evaluating neurochemistry (eg, magnetic resonance spectroscopy [MRS] studies). Extracted features are summarized in tables 1–3, and structural findings are visualized in supplementary result S4. There were not sufficient studies to perform a meta-analysis for any MRI modality due to a limited number of studies and high sample overlap between different studies.

**Table 1.** Outcomes of Included DTI and Structural MRI Studies Investigating the Difference Between Smoking and Nonsmoking Patients With SSD

Article	*n* Total (SS/NS/SC/NC) *n* Males	Diagnoses of Included Patients	Pack Years (SS/SC)	Cigarettes per Day (SS/SC)	Analysis	Outcome Measure	ROIs	Findings
DTI								
Cullen et al^23^	28/15/0/40 20/12/0/31	SCZ	NM	NM	Segmentation	ROI white matter FA	Whole-brain, cerebellum, brainstem, total cortical, frontal, temporal, parietal, occipital lobes	↓ FA in the whole brain, total cortical, and frontal lobes *SCZ vs controls* ↓ FA in the whole brain, total cortical, frontal, and occipital white matter regions *NC > NS > SS (not significant after IQ correction)*
Zhang et al^24^	32/14/48/20 30/10/45/15	SCZ	19.2 ± 19.4/18.2 ± 12.4	NM	Track-based spatial statistics	Whole-brain white matter FA	—	↓ FA in the left ATR/ALIC white matter regions in *SCZ vs controls* and *smokers vs nonsmokers → seeming additive effect* ↓ FA in the left and right UF/IFOF white matter regions in *SCZ vs controls* ↓ FA in the left frontal cortex white matter regions in *smokers vs nonsmokers*
Structural MRI								
Jørgensen et al^25^	250/256/48/189 131/130/34/99	SCZ, SAFD, BP	NA	14.2 ± 8.0/NA	Segmentation	ROI cortical thickness	Cingulate cortex, insula, DLPFC, OFC	↓ left rostral ACC and left insular cortex in *SS vs NS*
Ringin et al^26^	132/69/26/56 100/41/19/22	SSD	NA	NM	Segmentation	ROI GM volume, surface area, and cortical thickness	Hippocampus, amygdala, thalamus, STG, DLPFC, VLPFC, cingulate cortex, OFC, insula	↓ PCC thickness in *smokers vs nonsmokers* ↓ caudal ACC, pars opercularis, lateral OFC, medial OFC, pars orbitalis, pars triangularis, DLPFC, STG, and insula bilaterally cortical thickness in *SCZ vs controls* ↓ right DLPFC, pars orbitalis, and pars triangularis surface area in *SCZ vs controls* ↑ lateral OFC surface area in *SCZ vs controls*
Schneider et al^27^	53/59/0/77 37/44/0/47	SCZ	NM	NM	Segmentation	ROI GM volume and cortical thickness	Hippocampus, DLPFC	↓ right hippocampus and DLPFC in *SS vs NS and SS vs NC* ↓ left hippocampus in *SS* vs *NC* ↓ DLPFC and right amygdala *SS vs NC and NS* ↓ cortical thickness in V1 in *SS vs NS*
Tregellas et al^28^	14/18/2/48 10/11/NM/NM	SCZ	14.9 ± 15.0 (all smokers)	NA	VBM	Whole-brain GM volume	—	↑ STG and lateral PFC in *SS vs NS* ↓ OFC, insula, DLPFC, the STG, and PCC in *SCZ vs controls.*
Van Haren et al^22^	54/42/35/78 NM	SCZ, SFD	NA	23.8 ± 13.0/10.1 ± 7.0	Segmentation	ROI GM volume	Total brain, gray and white matter cerebrum, cerebellum, lateral/3rd ventricle	Significant associations between more pronounced cerebral gray matter decreases and a higher number of cigarettes smoked per day in *SCZ*
Yokoyama et al^29^	30/30/20/20 21/17/17/12	SCZ	23.30 ± 19.5/9.25 ± 6.8	NM	VBM	Whole-brain GM volume	—	↓ left PFC in *smokers vs nonsmokers* ↓ left PFC, left ACC, hippocampus, and insula in *SCZ vs controls* No interaction, but a seeming additive effect was found between diagnosis and smoking

Smoking-related values are mean ± SD. Pack years represent the cumulative exposure to smoking, calculated by multiplying the number of packs smoked per day by the number of years of smoking. *Note*: ACC, anterior cingulate cortex; ATR/ALIC, anterior thalamic radiation of the anterior limb of internal capsule; BP, bipolar disorder; DLPFC, dorsolateral prefrontal cortex; DTI, diffusion tensor imaging; FA, fractional anisotropy; FTND, Fagerström Test for Nicotine Dependence; GM, gray matter; IQ, intelligent quotient; MRI, magnetic resonance imaging; NA, not assessed; NC, nonsmoking controls; NM, not mentioned; NS, nonsmoking patients with SSD; OFC, orbito-frontal cortex; PCC, posterior cingulate cortex; PFC, prefrontal cortex; ROI, region-of-interest; SAFD, schizoaffective disorder; SC, smoking controls; SCZ, patients with SSD; SFD, schizophreniform disorders; SS, smoking patients with SSD; SSD, schizophrenia spectrum disorders; STG, superior temporal gyrus; UF/IFOF, uncinate fasciculus of the inferior fronto-occipital fasciculus; V1, the primary visual cortex; VLPFC, ventrolateral prefrontal cortex.

**Table 2.** Outcomes of Included Resting-State fMRI Studies Investigating the Difference Between Smoking and Nonsmoking Patients With SSD

Article	*n* Total (SS/NS/SC/NC) *n* Males	Diagnoses of Included Patients	Pack Years (SS/SC)	Cigarettes per Day (SS/SC)	Outcome Measures	Analysis	Findings		
Chen^30,a^	22/21/22/21 19/12/19/14	SCZ (5 FEP, 38 chronic)	20.01 ± 4.3/12.06 ± 3.3	23.09 ± 2.61/17.18 ± 1.86	FDG1 and 2	Principal component analysis on gradient pattern of extracted time-series features	↑ FDG1 in the bilateral OFC and the right posterior DAN in *SCZ vs controls* Significant smoking × diagnosis interaction for FDG1 in the bilateral PCC, the left inferior frontal gyrus, and the left temporal parietal junction, with more severe effects of smoking in controls than patients. Significant smoking × diagnosis interaction for FDG2 in the bilateral motor cortex and middle temporal gyrus with more severe effects of smoking in controls than patients. Positive PANSS symptoms negatively correlated to FDG1		
Fan et al^31,a^	22/27/22/21 19/12/19/14	SCZ (5 FEP, 44 chronic)	20.0 ± 4.31/12.1 ± 3.3	23.09 ± 2.61/17.18 ± 1.86	PFC chronnectomic density	Chronnectomic topological analysis using the sliding window method	↓ chronnectomic density in bilateral DLPFC *in SCZ* Significant smoking × diagnosis interaction on the right DLPFC chronnectomic density with a negative smoking effect *in controls*, and a positive smoking effect *in SCZ* No relationship between chronnectome density and symptom severity		
Liao et al^32,a^	22/25/22/21 19/10/19/14	SCZ (5 FEP, 42 chronic)	20.0 ± 4.3/12.1 ± 3.3	23.09 ± 2.61/17.18 ± 1.86	GC strength	GC analysis on unifying triple network dynamics	Smoking reduced negative GC strength from SN to DMN *in controls but not SCZ* Smoking increased positive GC strength from DMN to CEN *in SCZ but not controls* Significant smoking × diagnosis interaction in GC strength from the SN to the DMN with patients showing an active effect as a result of smoking in relation to controls		
Liao et al^33,a^	22/27/22/21 19/12/19/14	SCZ (5 FEP, 44 chronic)	20.0 ± 4.3/12.1 ± 3.3	23.09 ± 2.61/17.18 ± 1.86	Homotopic functional connectivity	Pearson’s correlation coefficients between symmetrical interhemispheric voxel time series	↓ interhemispheric FC between the bilateral dACC, thalamus, rolandic operculum, inferior frontal gyrus, paracentral lobule, subgenual cingulate cortex, and postcentral gyrus *in SCZ vs controls* ↓ interhemispheric FC in bilateral subgenual ACC in smokers vs nonsmokers Diagnosis × smoking interaction in the bilateral VLPFC, with ↑FC in *SS vs NS* and ↓FC *in SC vs NS* VLPFC interhemispheric FC negatively correlated with PANSS negative scores ↓ severe positive and negative symptoms *in SS vs NS*		
Liu et al^34^	21/21/0/21 19/15/0/14	SCZ	NA	17.1 ± 8.9/NA	iBA (whole-brain ALFF)	ANOVA with ROI-wise post hoc analyses	*NS vs NC* ↑ right caudate, MPFC ↓ right postCG	*SS vs NS* ↑ right caudate, left preCG, MPFC ↓ left caudate, right postCG	*SS vs NC* ↑ right STG, left preCG ↓ left caudate
Moran et al^35^	36/18/37/28 34/13/33/22	SCZ	20.8 ± 4.3/17.5 ± 2.3	19.5 ± 1.8/19.5 ± 1.2	FC	Seed-based FC with dACC as seed	↓ rsFC between the dACC and the right limbic, left posterior inferior temporal gyrus, right STG, left FG, right uncus, left parahippocampal, and right inferior parietal lobe *in SS and relatives vs SC* Significant smoking × diagnosis interaction in which smoking and diagnosis negatively influenced rsFC in the dACC-right limbic circuits.		
Ward et al^36^	18/0/17/0 9/0/9/0	SCZ (*n* = 15) and SAFD (*n* = 3)	15.1 ± 10.3/9.1 ± 5.5	16.3 ± 7.9/13.4 ± 3.8	FC	Multivariate pattern analysis of whole-connectome data	↑ correlation between daily cigarettes and FC of the parieto-occipital region to DMN *in SS* ↓ correlation between daily cigarettes and FC of the parieto-occipital region to DAN *in SS*		
Yang et al^37,a^	22/27/21/22 19/12/19/14	SCZ (5 FEP, 44 chronic)	20.0 ± 4.3/12.1 ± 3.3	23.09 ± 2.61/17.18 ± 1.86	Dynamic iBA (dynamic ALFF)	ANOVA	↑ dynamic iBA in the left SPG in *SCZ vs controls* Significant smoking × diagnosis interaction in the left DLPFC with increased dynamic ALFF *in SS vs NS*		

Smoking-related values are mean ± SD. Pack years represent the cumulative exposure to smoking, calculated by multiplying the number of packs smoked per day by the number of years of smoking. *Note*: ACC, anterior cingulate cortex; ALFF, amplitude of low-frequency fluctuation; CEN, central executive network; DAN, dorsal attention network; DLPFC, dorsolateral prefrontal cortex; DMN, default mode network; FC, functional connectivity; FDG, functional dynamics gradient; FEP, first-episode psychosis; FG, fusiform gyrus; fMRI, functional magnetic resonance imaging; GC, granger causality; iBA, intrinsic brain activity; MDMR, multidimensional matrix regression; NA, not assessed; NC, nonsmoking controls; NS, nonsmoking patients with SSD; PCC, posterior cingulate cortex; PFC, prefrontal cortex; postCG, postcentral gyrus; preCG, precentral gyrus; ROI, region-of-interest; rsFC, resting-state functional connectivity; SAFD, schizoaffective disorder; SC, smoking controls; SCZ, patients with SSD; SSD, schizophrenia spectrum disorders; SN, salience network; SPG, superior parietal gyrus; SS, smoking patients with SSD; STG, superior temporal gyrus; VLPFC, ventrolateral prefrontal cortex.

^a^Studies used a sample from the same dataset.

**Table 3.** Outcomes of Included Task-Based fMRI Studies Investigating the Difference Between Smoking and Nonsmoking Patients With SSD

Article	*n* Total (SS/NS/SC/NC) *n* Males	Diagnoses of Included Patients	Pack Years (SS/SC)	Cigarettes per Day (SS/SC)	Task	Outcome Measure	Findings
Friedman et al^38^	6/6/4/4 5/5/2/2	SCZ and SAFD	40.9/25.2	NM	Breath-hold task, visual activation task	% BOLD-signal change, activation volume, time-to-peak, time-to-trough	*Activation task* ↑ 22% larger signal change *in smokers vs nonsmokers* ↓ 39.7% reduction in activation volume *in SC vs NC* ↓ 24% reduction in activation volume *in SS vs NS* ↑ 4.3% increase in mean time to trough *in smokers vs nonsmokers* Significant smoking × diagnosis interaction for mean time to peak (fast time-to-peak in NS and a delayed time-to-peak in SS, relative to controls) *Breath-hold task* ↑50% larger signal change *smokers vs nonsmokers* ↑40% larger signal change *in SCZ vs controls*
Leyba et al^39^	15/15/0/15 13/11/0/9	SCZ	16.9 ± 13.0/—	NM	Auditory-motor task	% BOLD-signal change, volume of activation	No significant findings
Moran et al^40^	20/0/19/0 11/0/9/0	SCZ (*n* = 17) and SAFD (*n* = 3)	15.1 ± 10.8/9.2 ± 5.2	15.7 ± 8.0/13.1 ± 3.8	Smoking cue task	% BOLD-signal change	↓ activation for neutral > smoking in the right anterior frontal midline, right posterior frontal midline, and left frontal midline region *in SS vs SC*
Potvin et al^41,a^	18/0/24/0 14/0/15/0	SCZ (*n* = 14) and SAFD (*n* = 4)	NA	18.8 ± 5.4/20.5 ± 5.5	Smoking cue task	% BOLD-signal change	↑ activation for craving > neutral in the right caudate, bilateral vmPFC *in SS* ↓ activation for craving > neutral in the left lingual gyrus *in SS* ↑ activation for craving > neutral in the right angular gyrus, the anterior and posterior cingulate gyri, and the left superior frontal gyrus *in SC* ↓ activation for craving > neutral in the right lingual gyrus *in SC* right vmPFC activity correlated to cue-elicited cravings in *SS* ↑ activation for craving > neutral in right and left vmPFC *in SS vs SC*
Potvin et al^42,a^	21/0/23/0 16/0/15/0	SCZ (*n* = 18) and SAFD (*n* = 3)	NA	19.0 ± 5.2/20.3 ± 5.6	Aversive smoking cue task	FC	↓ FC for aversive tobacco > neural from the dmPFC to the amygdala *in SS*
Potvin et al^43,a^	18/0/27/0 NM	SCZ (*n* = 14) and SAFD (*n* = 4)	NA	18.8 ± 5.4)/20.5 ± 5.5	Smoking cue task	FC	↑ FC craving > neural between right nAC and the right middle temporal gyrus and precuneus, and between the left nAC and the left middle temporal gyrus, the right precuneus, and the left lateral occipital cortex *in SS vs SC* + correlation between the left nAC and left middle temporal gyrus FC and cigarette cravings *in SS and SC*

Smoking-related values are mean ± SD. Pack years represent the cumulative exposure to smoking, calculated by multiplying the number of packs smoked per day by the number of years of smoking. *Note*: BOLD, blood oxygen level dependent; dmPFC, dorsomedial prefrontal cortex; FC, functional connectivity; fMRI, functional magnetic resonance imaging; NA, not assessed; nAC, nucleus accumbens; NC, nonsmoking controls; NM, not mentioned; NS, nonsmoking patients with SSD; PFC, prefrontal cortex; SAFD, schizoaffective disorder; SC, smoking controls; SCZ, patients with SSD; SN, saliency network; SS, smoking patients with SSD; SSD, schizophrenia spectrum disorders; vmPFC, ventromedial prefrontal cortex.

^a^Studies used a sample from the same dataset.

### Structural Neuroimaging Studies

Eight studies investigated brain morphology changes in relation to smoking and schizophrenia, examining gray matter volume,^22,26–29^ cortical thickness,^25–27^ white matter integrity,^23,24^ and cortical surface area^26^ (table 1). Most studies conducted a region-of-interest analysis,^22,23,25–27^ and few a whole-brain analysis.^24,28,29^ Patients with SSD showed widespread gray matter reductions, primarily affecting the prefrontal cortex (PFC),^26–29^ superior temporal gyrus (STG),^28^ insula,^26,28,29^ anterior cingulate cortex (ACC),^26,29^ posterior cingulate cortex (PCC),^28^ hippocampus,^27,29^ and amygdala.^27^ Smoking was associated with reductions in the gray matter of the PFC,^27,29^ insula,^25^ ACC,^25^ PCC,^26^ hippocampus,^27^ and amygdala.^27^ Results are visualized in supplementary result S4.

The most frequently reported region in which significant differences were found was the PFC. Four studies observed a decrease in PFC volume and cortical thickness associated with schizophrenia^26,28^ or both schizophrenia and smoking,^27,29^ indicating a possible additive decrease in volume in smoking SDD patients. However, 3 studies specifically investigated the interaction effects of smoking status and schizophrenia on PFC structure and found no significant interactions.^22,26,29^ Moreover, another study reported a larger PFC volume in smoking compared with nonsmoking patients.^28^ The insula was also consistently reported, with significant decreases in gray matter volume and cortical thickness attributed to schizophrenia,^26,28,29^ and to smoking for cortical thickness.^25^ Other statistically significant gray matter reductions in relation to both smoking and schizophrenia were observed in the ACC,^25,26,29^ hippocampus,^27,29^ and amygdala.^27^

Both DTI studies reported an overlap of the pathology between schizophrenia and smoking-related phenotype.^23,24^ They showed that smoking patients with SSD had lower fractional anisotropy (FA) values than nonsmoking patients. Cullen et al found that patients had lower FA values in total brain, total cortical brain, and frontal lobes white matter compared with controls, also after intelligent quotient (IQ) correction. Smoking patients had lower FA values than both nonsmoking patients and controls in total brain, total cortical, frontal, and occipital white matter regions, but these differences became insignificant after adjusting for IQ.^23^ A more comprehensive whole-brain analysis revealed that reduced FA in the brain tract connecting the thalamus/striatum with frontal cortical regions, specifically the left anterior thalamic radiation of the anterior limb of the internal capsule, was independently and additively associated with both smoking and schizophrenia.^24^ Noteworthy, FA was not significantly associated with smoking-related measures (ie, Fagerström Test for Nicotine Dependence [FTND] score or pack years).

The only longitudinal study demonstrated that while schizophrenia was linked to a decline in overall gray matter volume, only heavy smokers (>more than 25 cigarettes per day), showed a decline in gray matter volume.^22^ Unfortunately, smoking-related measures were only collected at follow-up, thereby making it impossible to monitor shifts in participants’ smoking habits throughout the 5-year period.

### Functional Neuroimaging Studies

#### Resting-State fMRI

Eight resting-state fMRI studies investigated resting-state brain measures in relation to smoking and schizophrenia (table 2).^30–37^ Noteworthy, 5 of these studies (63%) used an almost identical sample from the same dataset.^30–33,37^ Studies employed a wide range of brain measures, including intrinsic brain activity (iBA),^34,37^ chronnectomic density,^31^ Granger causality strength,^32^ functional dynamics gradients (FDGs),^30^ and functional connectivity (FC).^33,35,36^ See supplementary material S2 for a detailed explanation of these measures. Six resting-state studies showed an interaction effect between smoking and SSD diagnosis.^30–33,35,37^ Additionally, 1 study identified overlapping regions (ie, right caudate, right postcentral gyrus, and medial PFC) related to both schizophrenia and smoking effects, but the study design did not allow for investigation of an interaction effect.^34^ Collectively, studies suggest interactions between smoking and schizophrenia within the PFC, and default mode network (DMN) and limbic system regions.

The most consistent finding across studies was that the activity of the PFC is affected by both smoking and schizophrenia.^31,33,34,37^ Specifically, 1 study identified an interaction effect between smoking and SSD diagnosis in the ventrolateral prefrontal cortex (VLPFC), where smoking increased interhemispheric FC (ie, connectivity between a brain region bilaterally) in patients and decreased interhemispheric FC in controls.^33^ Thus, smoking appears associated with distinct effects on neural connectivity in patients with SSD compared with controls. One study demonstrated an interaction effect in the dorsolateral prefrontal cortex (DLPFC), with decreased temporal dynamic iBA in patients compared with controls, and nonsmoking patients compared with smoking patients.^37^ Decreased temporal dynamic iBA suggests a decrease in the variability or oscillations of low-frequency brain activity. Another study showed an association between smoking and less deterioration of BA in the medial PFC of patients,^34^ suggesting a possible link to the restoration of typical spontaneous brain activity. Lastly, 1 study observed an interaction effect of chronnectomic density in the right DLPFC, with decreased chronnectomic density in smoking compared with nonsmoking controls, and increased chronnectomic density in smoking compared with nonsmoking patients.^31^ Higher chronnectomic density in a brain region indicates that there is a greater level of information integration and connectivity within that specific region. Furthermore, nonsmoking controls had significantly higher chronnectomic density compared with both smoking and nonsmoking patients, while smoking controls resembled both groups of patients. Thus, findings suggest a potential association between smoking and a preserving effect on disrupted prefrontal dynamics in schizophrenia. Of the abovementioned studies, Liao et al report that (1) VLPFC interhemispheric FC is negatively correlated with the Positive and Negative Syndrome Scale (PANSS) negative scores, and (2) smoking patients with SSD reported less severe positive and negative symptoms than nonsmoking patients.^33^ However, the other 3 studies report no relation between outcome measures and symptom severity.^31,34,37^

One study demonstrated a significant interaction between smoking and SSD diagnosis on intrinsic neural dynamics through 2 different FDGs. For FDG1, this was in the bilateral PCC, the left inferior frontal gyrus, and the left temporal parietal junction, and for FDG2 in the bilateral motor cortex and middle temporal gyrus.^30^ The effect of smoking was more severe in controls than in patients, and results suggest that smoking appears associated with distinct effects on patients compared with controls. The findings indicate that there is a disturbance in the neural dynamics of the DMN and salience attention network in schizophrenia smokers (FDG1) and there are alterations in the sensorimotor-association cortices transition (FDG2). Furthermore, in SSD smokers, it was found that the strength of FDG1 of the left PCC was negatively correlated with the PANSS total and positive scores. In other words, SSD patients showed a lower dynamics gradient with higher nicotine dependence, and this was associated with more symptoms.

Two studies investigating network FC associate both smoking and schizophrenia to the DMN.^32,36^ Ward et al^36^ found that in patients with SSD who smoked more cigarettes a parieto-occipital region was increasingly likely to be part of the DMN instead of belonging to the dorsal attention network (DAN). Conversely, as SSD patients smoked fewer cigarettes, the likelihood of the parieto-occipital region being a part of the DAN increased. This effect was not seen in controls. Liao et al reported an association between smoking and decreased connectivity between the salience network, composed of the anterior insula and dorsal ACC (dACC), and the DMN in controls, but this was not seen in patients.^32^ Moreover, smoking patients also displayed increased connectivity of the DMN to the central executive network, but this was not seen in controls. Thus, the results of Ward et al^36^ suggest a negative association between chronic smoking and DMN FC in patients, whereas Liao et al^32^ suggest a positive association between chronic smoking and the regulation of DMN in patients.

Two studies found that smoking and schizophrenia have an additive adverse effect on ACC connectivity, indicating a possible shared neurobiological mechanism.^33,35^ One study found reduced FC in a dACC-right limbic circuit in smoking patients compared with smoking controls.^35^ Noteworthy, this effect was also found in smoking first-degree relatives of patients with SSD compared with smoking controls, indicating that the genetic susceptibility to schizophrenia may play a role in the reductions in resting-state FC between the dACC and right limbic circuits, shared in schizophrenia and nicotine addiction. Additionally, the authors demonstrated a significant interaction between smoking and SSD diagnosis in which both smoking and diagnosis negatively influenced resting-state FC in the dACC-right limbic circuits.^35^ Furthermore, Liao et al^33^ demonstrated lower interhemispheric connectivity of the subgenual ACC both in relation to smoking and schizophrenia.^33^

In summary, smoking appears associated with distinct effects on neural dynamics in individuals with and without schizophrenia,^30–33,36^ shedding light on the complex neurobiological mechanisms underlying this interaction. Findings indicate a potential association between smoking and improvements in connectivity deficits linked to schizophrenia^34,37^ or a preserving effect of smoking on brain activity,^30–32,35^ while 2 studies suggested the existence of an additive negative effect.^33,35^

#### Task-Based fMRI

Six studies investigated task-dependent changes in fMRI in relation to smoking and schizophrenia^38–43^ (table 3). Noteworthy, 3 of these studies (50%) employed samples from the same dataset.^41–43^ See supplementary material S2 for information on the different tasks that were used.

Four studies employed smoking cue tasks.^40–43^ Smoking cue tasks are used to investigate the neural correlates of smoking-related cues (eg, pictures of cigarettes or people smoking) in the brain. Smokers with schizophrenia showed higher smoking-cue-induced activity in the ventromedial prefrontal cortex (vmPFC) compared with smoking controls,^41^ and increased smoking-cue-induced FC between the nucleus accumbens (nAC) and the middle temporal gyrus, precuneus, and the left lateral occipital cortex.^43^ Furthermore, right vmPFC activity was correlated to cue-elicited cravings in smoking patients but not smoking controls,^41^ and in both groups of smokers a positive correlation was observed between the left nAC and left middle temporal gyrus connectivity and cigarette cravings.^43^ Both studies show that smokers with schizophrenia have a heightened activity in the brain reward system in response to cigarette cues, indicating that cigarettes are more reinforcing and subjectively more valuable to them compared with smokers without schizophrenia. Furthermore, connectivity between the dorsal medial PFC and amygdala was reduced when viewing smoking-aversive images.^42^ This suggests that the dorsal medial PFC, involved in complex cognitive functions, inhibits the response of the amygdala to anti-smoking images in smokers with schizophrenia, leading to cognitive-affective dissonance and reduced awareness of the harmful consequences of tobacco smoking. Taken together, these studies imply that the reward system is more activated and interconnected with the DMN, indicating an enhanced subjective value of cigarettes. Simultaneously, there is impaired connectivity between the dorsal medial PFC and amygdala, suggesting disrupted processing of negative consequences associated with smoking, such as its health consequences. On the other hand, a fourth study on smoking cue reactivity in schizophrenia reported 3 clusters in which smoking patients with SSD showed less cue-induced activation than smoking controls (ie, right anterior frontal midline, right posterior frontal midline, and left frontal midline region).^40^ This suggests that increased response to smoking-related cues may not be the leading cause of increased smoking and low quitting rates in schizophrenia.

A small study (*n* = 4–6 per group) demonstrated a significant interaction effect between smoking and SSD diagnosis for the mean time to activation peak, with a faster blood oxygen level-dependent response peak in nonsmoking patients and a delayed time-to-peak in smoking patients, both relative to smoking and nonsmoking controls, in a simple visual activation task using checkerboard stimuli.^38^ Lastly, 1 study employing an auditory-motor task comparing smoking and nonsmoking patients and controls revealed no significant findings.^39^

### Risk of Bias Assessment of and Involvement Tobacco Industry in Included Studies

The NIH Quality Assessment Tool *for Observational Cohort and Cross-Sectional Studies* was used on all 22 studies (supplementary result S5). Among structural MRI studies, 5 were rated as high quality (ie, low risk of bias), 3 as fair quality (ie, fair risk or bias), and none as poor quality (ie, high risk or bias). Among resting-state fMRI studies, 7 were rated as high quality, and 1 as fair quality. Among task-based fMRI studies, all 6 studies were rated as high quality.

We found that 1 study^29^ was directly funded by the tobacco industry by the Smoking Research Foundation,^44^ and 2 further studies were directly funded by pharmaceutical corporations.^43,41^ The authors of 4 studies received lecture or consultancy fees from pharmaceutical corporations or were otherwise involved with the pharma industry,^22,26,27,36^ one of which was not supported by public or departmental funds, but multiple pharmaceutical companies sponsored all authors.^22^ None of the here reviewed studies in which a potential conflict of interest was identified reported evidence favoring the self-medication hypothesis.^17^ Conversely, functional imaging studies that observed favorable results in relation to smoking and FC abnormalities were conducted based on public and departmental funds.

## Discussion

Our review integrated findings from 22 functional or structural MRI studies on the interrelationship between chronic tobacco smoking and schizophrenia. Importantly, all but 1 study had a naturalistic cross-sectional design, thus these studies cannot untangle a predisposition that smoking patients have (eg, less deviant resting-state activity from controls) as opposed to an actual effect of smoking (eg, tobacco normalizing resting-state activity of patients).

The reviewed structural studies show reductions in PFC,^26–29^ STG,^28^ insula,^25,26,28,29^ ACC,^25,26,29^ PCC,^26,28^ hippocampus,^27,29^ and amygdala^27^ gray matter in relation to smoking and schizophrenia (table 1 and supplementary result S4). Existing literature on brain morphology changes related to schizophrenia and smoking separately supports the notion that both are related to widespread gray matter reductions.^45–48^ The overlap in brain regions structurally affected by both smoking and schizophrenia suggests shared neurobiological pathways. In terms of resting-state activity, an interaction effect between smoking and schizophrenia was found in the PFC,^31,33,34,37^ DMN,^32^ and limbic system^35^ regions (table 2). Results mainly suggest a potential association between smoking and improvements in connectivity deficits linked to schizophrenia^34,37^ or a preserving effect of smoking on brain activity.^30–32,35^ However, 2 studies also suggested the existence of an additive negative effect.^33,35^ Overall, smoking appears associated with distinct effects on neural dynamics in individuals with and without schizophrenia.^30–33,36^ Task-based fMRI studies show smoking patients have smoking-cue-induced hyperactivity in the PFC and from the nAC to the DMN.^43,41^ Furthermore, connectivity between the PFC and amygdala is reduced when viewing smoking-aversive images.^42^ Lastly, a fourth study on smoking cue reactivity in schizophrenia reports 3 clusters in which smoking patients showed less cue-induced activation than smoking controls. Together, task-based fMRI results suggest that the brain reward system might play a role in the schizophrenia-tobacco smoking co-occurrence. Studies point to a unique neural activation pattern in smokers with schizophrenia,^38,41–43^ characterized by increased reward system sensitivity but also by a disconnection in the neural pathways that normally mediate the cognitive and affective processing of the negative consequences of smoking. This combination of findings highlights the multifaceted disruption in neural networks in schizophrenia smokers, which might contribute to their heightened vulnerability to tobacco addiction.

**Fig. 2.**
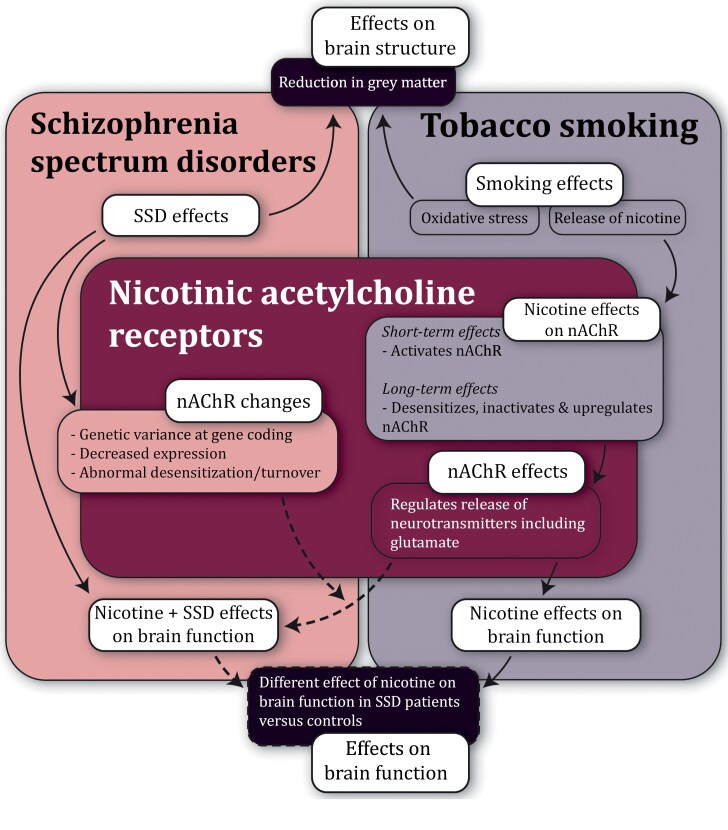
Theoretical framework presenting the hypothesized framework on chronic tobacco smoking, brain structure, and function in patients with SSD. This figure highlights the hypothesized framework explaining the differential association of smoking on neural dynamics in patients with SSD vs controls. Dashed arrows/boxes indicate hypothesized effects. Structural studies show that smoking is associated with negative effects on gray matter in both controls and patients, potentially worsening SSD-related abnormalities. Functional findings show that smoking appears associated with distinct effects on neural dynamics in patients vs controls, posing a possible schizophrenia-specific neurobiology of tobacco addiction. A central role of nAChRs is hypothesized, with genetic variations and expression changes in SSD on the nAChR, which in turn affect neurotransmitter release and thus the brain’s response to nicotine, diverging from controls. This suggests that SSD-related neurobiological alterations at the nAChR level may explain the differential effects of nicotine on the psychotic vs nonpsychotic brain. *Note*: nAChR, nicotinic acetylcholine receptor; SSD, schizophrenia spectrum disorders.

Our review reveals a contrasting impact of smoking on the brain. Structural results confirm the negative consequences of smoking on gray matter in both patients with schizophrenia and controls, and studies point toward the exacerbation of structural abnormalities associated with the disorder by smoking. Functional resting-state findings, however, are considered mixed; Studies mostly show less connectivity deficits in smoking compared with nonsmoking patients.^34,37^ However, it is important to consider that the results of 5 of these studies (83%) stem from the same sample, and studies in other samples suggest an additive negative effect^35^ of smoking and schizophrenia, or that smoking promotes DMN hyperactivity^36^ which is generally seen in schizophrenia patients. Interestingly, although results are mixed, smoking appears associated with distinct effects on neural dynamics in patients vs controls,^30–33,36^ advocating for a different neural foundation of tobacco addiction in patients compared with controls. Task-based studies also point toward this, as results mostly imply a unique neural activation pattern in smokers with schizophrenia.^38,41–43^ It is, however, again important to note that these results stem from the same sample. A schizophrenia-specific neural foundation for the pathophysiology of tobacco addiction in patients is also endorsed by the findings that patients extract higher levels of nicotine from their cigarettes compared with control smokers,^49^ and that it may not be effective to use the same brain stimulation target (ie, the DLPFC) in patients as in controls to successfully treat nicotine dependence.^50^ The nicotinic acetylcholine receptor (nAChR) may play a key role in explaining functional differences observed between smoking patients and controls. Both tobacco smoking and patients with schizophrenia exhibit shared genetic variance at the Cholinergic Receptor Nicotinic Alpha gene cluster, which encodes for nAChRs.^13,51^ This shared genetic variation could independently contribute to the risk of smoking and schizophrenia, or increase the risk of smoking, which could then contribute to the later development of schizophrenia. The primary addictive component in tobacco, nicotine, exerts its effects on the brain by activating nAChRs. Patients with schizophrenia show decreased nAChR expression compared with controls in postmortem brain,^52^ and abnormal assembly or trafficking of these receptors in schizophrenia is implied.^53^ Importantly, these receptors regulate the release of many neurotransmitters including glutamate,^54,55^ which is an excitatory neurotransmitter essential for proper brain function. This reduced expression can alter the normal functioning of these receptors, leading to disruptions in neurotransmitter release. Furthermore, nAChRs are desensitized,^56^ inactivated, and upregulated^57^ due to chronic tobacco exposure, and it is suggested that patients show abnormal desensitization or turnover of α4β2 nAChRs.^52^ These alterations in receptor dynamics could further disrupt neurotransmitter regulation and contribute to the observed differences in response to nicotine. Thus, disease-related alterations in neurobiology at nAChR level may account for the observed divergent response of the brain of subjects with SSD to nicotine compared with the brain of subjects without a psychotic disorder. Figure 2 illustrates the theoretical framework of the hypothesized relationship between chronic tobacco smoking and SSD in terms of brain structure and function.

Intriguingly, when the FC observed in smoking patients is more similar to controls compared with nonsmoking patients, this does not necessarily correspond to improvements in symptomatology,^31,34,35,37^ which suggests a more complex and dissociative relationship between functional and clinical outcomes. The commonly believed self-medication hypothesis proposes that patients with SSD turn to smoking cigarettes to alleviate their cognitive deficits or symptoms. Six (75%) resting-state studies show that smoking patients resemble controls more or show a preservation effect of smoking in terms of activation, and the authors state this is in line with the self-medication hypothesis. However, 4 of these (67%) studies fail to show that their findings are related to fewer symptoms or cognitive problems in smoking patients.^31,34,35,37^ Therefore, it is unclear how smoking patients benefit from their potentially normalized FC. It is important to emphasize that these findings are based on cross-sectional observational studies, without investigating acute nicotine effects or any potential short-term benefits of smoking.

MRI studies investigating the acute effects of nicotine on the brain of patients with schizophrenia typically employ task fMRI alongside acute nicotine administration. Almost all studies suggest potential enhancing or even “reversing” effects related to nicotine, both in task performance as well as activity-associated brain regions. These effects encompass various cognitive domains, including attention,^58,59^ cognitive control,^60^ smooth pursuit eye movements,^61–65^ working memory,^64^ and sensorimotor gating.^66^ Two studies found similar positive effects in regard to resting-state connectivity involving the salience network^67,68^ and DMN.^36^ This is unsurprising, as acute nicotine administration itself is associated with positive effects on cognition by increasing activity in multiple brain regions.^69^ However, the cognitive benefits observed acutely may not persist with chronic nicotine use, as evidenced by a meta-analysis where chronic smoking was found to be associated with impairments across multiple cognitive domains in patients with psychosis.^70^ Furthermore, in patients with schizophrenia, no significant alterations were observed for global cognitive test performance with smoking cessation, abstinence, or resumption.^71^ It is important to acknowledge the potential positive effects of pure nicotine, whereas the long-term presence of other toxic substances in tobacco can induce inflammation and oxidative stress, potentially resulting in gray matter loss and lack of cognitive improvement. Research using transcranial magnetic stimulation^50^ or nAChR agonists^72^ might provide insight into the distinctions between tobacco and nicotine effects. This holds promise for elucidating a causal relationship between nicotine and cognitive function.

Studies were of fair to good quality (supplementary figure S3). However, several limitations should be acknowledged. First, all but 1 study exhibited a moderate risk of bias due to their cross-sectional nature, therefore examining smoking behavior and MRI outcomes concurrently and only once. Noteworthy, the only longitudinal study assessed exposure only at follow-up,^22^ neglecting possible changes in smoking habits. Cross-sectional studies do not allow for establishing temporal relationships or causality, and hinder the understanding of the impact of prolonged smoking on disease progression. Longitudinal studies help distinguish between preexisting anatomical and physiological differences and the (long-term) effects of smoking. In addition, if there is a causal effect between smoking and schizophrenia the impact of smoking may differ across various stages of the disease. Longitudinal and exposure-dependent research, differentiating between novice smokers vs long-term smokers and early stages of psychosis (eg, ultra-high risk for psychosis or first-episode psychosis patients) vs chronic schizophrenia, could help to properly disentangle the addiction effect from the possible correction of smoking to the neural dynamics in patients with schizophrenia.

Second, the number of included studies was small, there was a large variety in outcome measures, and almost all (86%) included studies had small to moderate sample sizes (<30), while only 1 study performed power calculations.^43^ This limits the statistical power and generalizability of the findings. In addition, it is crucial to take into account that the majority of the resting-state (63%) and task-based (50%) studies employed data from the same samples for their analyses. The interdependence of results stemming from the same sample could mistakably enhance the significance of findings, or limit the generalizability of conclusions. It is therefore important that these results are replicated in independent studies.

Third, it is challenging to disentangle the complex relationship between smoking and functional imaging outcomes. The dynamic nature of functional brain changes complicates the interpretation of whether the observed alterations are attributable to the chronic, acute, or withdrawal effects of tobacco, of which the latter 2 affect brain function in the short term.^14,73^ In contrast, structural MRI studies offer a more stable perspective, as structural differences typically take longer to manifest. This underscores the importance of considering the temporal dynamics and nuanced effects of nicotine in schizophrenia to better understand their interaction and their impact on the observed neural alterations.

Fourth, covariate inclusion varied among studies, with 5 studies not using any covariates.^24,28,35,41,42^ This could have led to residual confounding, posing a limitation to a reliable interpretation of the results. Three reviewed studies observed that smoking patients with SSD had lower cognitive capability than nonsmoking patients,^28^ although 2 accounted for this in additional analyses.^25,27^ This aligns with existing reports suggesting that individuals with lower IQ scores are more prone to smoking, possibly contributing to the higher smoking prevalence among patients with SSD.^74^ Only 9 studies (41%) controlled for a measure of intelligence, and 1 demonstrated that the variation in IQ accounted for the effect of smoking.^23^ Furthermore, none of the studies accounted for cannabis use in their analyses, even though cannabis use and tobacco smoking are highly correlated.^75^ Therefore, it is crucial to take into account potential confounders such as IQ and cannabis use in future research.

Fifth, it is important to consider that most patients with SSD in these studies used antipsychotic medication, making it challenging to disentangle effects by disease progression and medication exposure. Studies have frequently reported the effects of antipsychotic medication on brain structure and striatum and DMN function, with different effects for different antipsychotics and follow-up periods.^76^ Furthermore, as both nicotine and antipsychotic medication target dopamine systems, smoking may have implications for the effects of antipsychotic medications and vice versa. For example, chronic exposure to antipsychotic drugs affects nAChR expression in different brain regions in rats.^77,78^ Consequently, the effects on nAChRs in smokers with schizophrenia, which could potentially impact brain structure or function, might be a result of an interaction between nicotine and antipsychotic medications. On that note, smoking increases the metabolism of antipsychotic medication, thereby lowering medication concentration in the blood.^79^ Despite the potential confounding effect of antipsychotic medication, the 10 studies that did consider medication use in their analyses did not find an influence of medication on the results.^25,31–33,37,39–43^

Finally, it is worth noting that in all the studies reviewed here where a potential conflict of interest was identified, none of them presented evidence in favor of the self-medication hypothesis.

For future research, longitudinal and exposure-dependent studies are warranted to distinguish between inherent anatomical and physiological differences and the long-term effects of smoking in schizophrenia, particularly across different stages of smoking and psychosis. Such research is key to understanding the complex relationship between smoking and schizophrenia, and how this interaction may vary across different stages of the disease. Unimodal functional and structural research both have their strengths and limitations, complementing each other. Structural MRI reveals anatomical alterations associated with smoking and schizophrenia, offering insights into anatomical neurobiological consequences of the co-occurrence but lacks the ability to capture dynamic changes related to brain function. In contrast, fMRI dynamically maps brain activation at rest and during cognitive tasks, yet is more susceptible to confounding factors. Therefore, it is important that future functional research considers the short-term effects of acute and withdrawal effects of nicotine on the brain. Furthermore, currently lacking research on neurochemistry such as MRS is crucial to achieve a comprehensive understanding of the interplay between brain structure and function. Neurochemistry serves as a crucial bridge between brain structure and function, since communication between 2 anatomically connected regions occurs via synaptic transmission of neurotransmitters such as glutamate and acetylcholine. Finally, but most importantly, unimodal results should be integrated for a more complete, multimodal view.

This review underscores the intricate relationship between chronic tobacco smoking and schizophrenia. While structural studies demonstrate gray matter reductions in smoking SSD subjects over and above the reductions found in schizophrenia, functional studies present a more complex picture but suggest that smoking is associated with distinct effects on neural dynamics in individuals with and without schizophrenia. This implies a distinct neurobiological mechanism for tobacco addiction in those with schizophrenia, possibly attributed to differences at the nAChR level. The limited number of studies with varied outcome measures and a high amount of cross-sectional studies, along with the potential of the exaggerated significance of findings by repeated use of the same sample, require further research and replication in independent studies, especially as the currently limited samples prohibit meta-analysis. Furthermore, we advocate for more neurochemical, longitudinal, and exposure-dependent studies, and analysis of research in a multimodal manner. Ultimately, by gaining deeper insights into the underlying neurobiological basis of this co-occurrence we could find and address factors that contribute to the susceptibility of patients to smoking more effectively. This approach has the potential to improve the development of targeted interventions to improve smoking cessation, clinical outcomes, and life expectancy of patients with schizophrenia.

## Supplementary Material

Supplementary material is available at https://academic.oup.com/schizophreniabulletin/.

sbae088_suppl_Supplementary_Materials
