# Supplementary material for: The Association Between Chronic Tobacco Smoking and Brain Alterations in Schizophrenia: A Systematic Review of Magnetic Resonance Imaging Studies
Source: Schizophr Bull. 2024 Jun 2;51(3):608–24. doi: 10.1093/schbul/sbae088 (PMC12061661; doi:10.1093/schbul/sbae088)
Supplement: sbae088_suppl_Supplementary_Materials [file sbae088_suppl_supplementary_materials.docx]

**Supplement -** **The association between chronic tobacco smoking and brain alterations in schizophrenia: A systematic review of magnetic resonance imaging studies**

Supplementary materials

- S1 Search syntax and outcomes per search engine *Page 2*
- S2 Explanations of different MRI techniques. *Page 3*
- S3 Data Extraction and Assessment *Page 5*

Supplementary results

- S4 Regions with significant grey matter changes related to smoking and *Page 6*

schizophrenia in structural MRI studies

- S5 Outcomes of NIH quality assessment for all included studies *Page 7*

**S1 Search syntax and outcomes per search engine**

|  | **Search Terms** | **Hits** |
| --- | --- | --- |
| **PsychINFO** | |  |
| 1 | exp schizophrenia/ | 99,282 |
| 2 | (schizophreni* or severe mental illness or psychos*).ab,jx,id,ti. | 313,908 |
| 3 | "3213".cc | 63,160 |
| 4 | 1 or 2 or 3 | 317,247 |
| 5 | Exp smoke/ | 1,008 |
| 6 | Exp nicotine/ | 12,661 |
| 7 | (nicotin* or smoke* or smoking or tobacco* or cigarette*).ab,jx,id,ti. | 84,629 |
| 8 | 5 or 6 or 7 | 84,663 |
| 9 | Exp Brain/ | 296,981 |
| 10 | Exp Magnetic Resonance Imaging | 55,324 |
| 11 | (MRI or magnetic resonance imaging or magnetic resonance spectroscopy or connectivity or volume or fMRI or neuroimaging).ab,jx,id,ti | 207,066 |
| 12 | "0100".md | 78,868 |
| 13 | 9 or 10 or 11 or 12 | 434,751 |
| **14** | **4 and 8 and 13** | **506** |
| **Web of Science** | |  |
| 1 | TS=(schizophreni*) OR TS=(psychos*) OR TS=("severe mental illness") | 426,089 |
| 2 | TS=(nicotin*) OR TS=(smoke*) OR TS=(smoking) OR TS=(tobacco*) OR TS=(cigarette*) | 590,417 |
| 3 | TS=(MRI) OR TS=("magnetic resonance imaging") OR TS=(connectivity) OR TS=(volume) OR TS=(fmri) OR TS=(neuroimaging) OR SU=("Radiology, Nuclear Medicine & Medical Imaging") | 2,955,733 |
| **4** | **1 AND 2 AND 3** | **470** |
| **PubMED** | |  |
| 1 | (schizophrenia[MeSH Terms]) OR (schziophreni*[Title/Abstract]) OR (severe mental illness[Title/Abstract]) OR (psychos*[Title/Abstract]) | 312,840 |
| 2 | (smoke[MeSH Terms]) OR (nicotine[MeSH Terms]) OR (nicotin*[Title/Abstract]) OR (smoke*[Title/Abstract]) OR (smoking[Title/Abstract) OR (tobacco*[Title/Abstract]) OR (cigarette*[Title/Abstract]) | 481,462 |
| 3 | (Brain[MeSH Terms]) OR (Magnetic Resonance Imaging[MeSH Terms]) OR (MRI[Title/Abstract]) OR (magnetic resonance imaging[Title/Abstract]) OR (magnetic resonance spectroscopy[Title/Abstract]) OR (connectivity[Title/Abstract]) OR (volume[Title/Abstract]) OR (fmri[Title/Abstract]) OR (neuroimaging[Title/Abstract]) | 2,546,291 |
| **4** | **1 AND 2 AND 3** | **630** |
| **BIOSIS** | |  |
| 1 | schizophrenia.ds. | 55,953 |
| 2 | (schizophreni* or "severe mental illness" or psychos*).ab,jx,ti. | 100,197 |
| 3 | 1 OR 2 | 109,896 |
| 4 | (nicotin* or smoke* or smoking or tobacco* or cigarette*).ab,jx,ti. | 175,402 |
| 5 | Magnetic Resonance Imaging.mq. | 90,908 |
| 6 | (MRI or magnetic resonance imaging or magnetic resonance spectroscopy or connectivity or volume or fMRI Or neuroimaging).ab,jx,ti | 439,939 |
| 7 | 5 OR 6 | 476,835 |
| **8** | **3 AND 4 AND 7** | **118** |

**S2 Explanation of different MRI techniques and measures**

Structural neuroimaging studies

Structural neuroimaging studies typically use T1-weighted images to be able to detect alterations in brain volume, cortical thickness and/or surface area. Structural neuroimaging studies, in addition to standard T1-weighted MRI, also encompass diffusion tensor imaging (DTI). DTI is a non-invasive imaging technique that measures the diffusion of water molecules in tissue. In the brain, the diffusion of water is influenced by the orientation of white matter fibres. One of the most frequently used measures in DTI is fractional anisotropy (FA), which quantifies the strength of directionality of the diffusion. By analysing the diffusion patterns of water molecules, DTI can provide information about the directionality and integrity of white matter tracts.

Functional neuroimaging studies

Imaging the brain at rest without explicit task engagement picks up on low-frequency fluctuation across spatially distant areas. The co-occurrence of activity across brain regions gives insight into the brain's intrinsic functional organization and allows for example investigation of so-called resting-state networks.

*Resting-state brain activity measures*

1. Functional Connectivity

Functional connectivity measures the degree of statistical association or correlation between the activity of different brain regions.

2. Intrinsic Brain Activity

Intrinsic Brain Activity (iBA) refers to the spontaneous activity in the brain that occurs when a person is at rest, such as during resting-state fMRI. Temporal dynamic iBA refers to the variation or changes in the intrinsic activity of the brain over time.

3. Chronnectomic density

Chronnectomic density measures time-varying changes of connectomic density to evaluate the dynamic integration function of a region, rather than its intrinsic activity (Fan et al., 2020). Thus, it assesses dynamic functional connectivity patterns. In other words, it is a measure to assess the density of functional connections between different regions over time, helping us understand how different parts of the brain work together dynamically. Higher chronnectomic density in a brain region indicates that there is a greater level of information integration and connectivity within that specific region.

4. Granger causality strength

Granger causality analysis can be used to investigate dynamics and directionality of the BOLD signal in cortical circuits (Liao et al., 2011). The GC strength is then how strong and reliable the causal relationship between two brain regions is. It's measured by examining the statistical significance of the past activity in one region's time series in predicting the future activity in another region's time series.

5. Functional Dynamics Gradients

Neural activity or neural dynamics, leads to, due to the brain its hierarchical organisation, the formation of functionally distributed networks. These dynamics can be used to compare temporal similarities and to identify functional dynamical hierarchies, referred to as gradients (Shafiei et al., 2020). Thus, Functional dynamics gradient refers to the spatial organization of neural activity patterns based on the temporal features of brain regions' time series. It is a comprehensive characterization of the rich temporal patterns of neural activity, describing the distribution, autocorrelation structure, stationary properties, entropy, predictability, and model fits of the time series. Chen (2023) describe two functional dynamics gradients (FDGs) (Chen, 2023). FDG1 characterizes the ventromedial-dorsolateral axis in the brain's topological organization, primarily focusing on capturing temporal coordination and autocorrelation patterns in timeseries data. It helps elucidate how specific brain regions synchronize and influence each other's activity over time, contributing to complex cognitive functions. FDG2 spans from unimodal-to-transmodal regions and describes the shape and distribution of timeseries amplitudes. It is associated with the canonical functional gradient, intra-cortical myelin, and cortical thickness, suggesting a role in both functional and structural aspects of brain organization. These gradients are used for comprehensively characterizing the rich temporal patterns of neural activity and mapping the topological organization of brain functional dynamics.

Task-based fMRI

Two common types of tasks studied in the literature are smoking-cue tasks and sensory activation tasks. Smoking-cue tasks are used to investigate the neural correlates of smoking-related cues (e.g. pictures of cigarettes or people smoking) in the brain. The sensory activation task involves the presentation of auditory or visual stimuli. Tasks were employed in a block design, with the signal averaged across a "stimulus on" and "stimulus off" block, or an event-related design, where stimulus conditions are interleaved and the signal acquired per trial. By calculating the signal change between the presence and absence of the smoking cue or sensory stimuli, a better understanding of the neural mechanisms that drive smoking behaviour or sensory processing in different populations can be gained.

**S3 Data extraction and assessment**

Categories, items and domains for the extraction of data are presented in the below. The definitions of key limitations and strengths adapted for the purpose of this review based on COBIDAS.

| **Category** | **Items/Domains** |
| --- | --- |
| Publication | Article id, author and date of publication, journal |
| Study population | No. of subjects per group, mean and SD age per group, no. of females per group, IQ per group (alternatively years of education), criteria to be classified as smoker or non-smoker, smoking severity (pack-years, cigarettes per day, FTND score), |
| Clinical characteristics | Included diagnoses, medication use information, age of disease onset |
| Study design | Inclusion and exclusion criteria (SSD and controls), nicotine abstinence before MRI scan, ethical compliance, MRI technique |
| MRI acquisition | Image sequence (T1, T2, ASL, etc), task (resting-state, or type of task), MRI field strength, TR/TE/flip angle, voxel resolution (of functional sequences if applicable), scan duration |
| Preprocessing | Preprocessing pipeline, spatial smoothing FWHM, motion correction method, normalization space, regions of interest, metric |
| Group analysis | Contrast, covariates, multiple comparison methods, reporting of coordinates |
| Results | Results summary, additional analysis, differences between groups in clinical/cognitive outcomes, interpretation summary, mean + SD of findings for structural studies |
| Funding / open access | Funding, involvement of the pharma industry, pre-registration of the protocol, public sharing of data |
| Quality assessment, strengths and limitations | Key limitations (observational/naturalistic study, limited sample size (<30/per group), covariate inclusion in analyses, poorly reported head motion correction, too stringent/loose multiple comparison correction, lack of non-smoking controls group, not accounting for medication effects, not accounting for disease progression) |

Abbreviations: ASL, Arterial spin labelling; COBIDAS, Committee on Best Practice in Data Analysis and Sharing; FTND, Fagerström Test for Nicotine Dependence; FWHM, full-width at half-maximum; IQ, intelligent quotient; MRI, magnetic resonance imaging; no, number of; SD, standard deviation; SSD, schizophrenia spectrum disorder; TE, echo time; TR, repetition time; T1, T1-weighted image; T2, T2-weighted image.

**
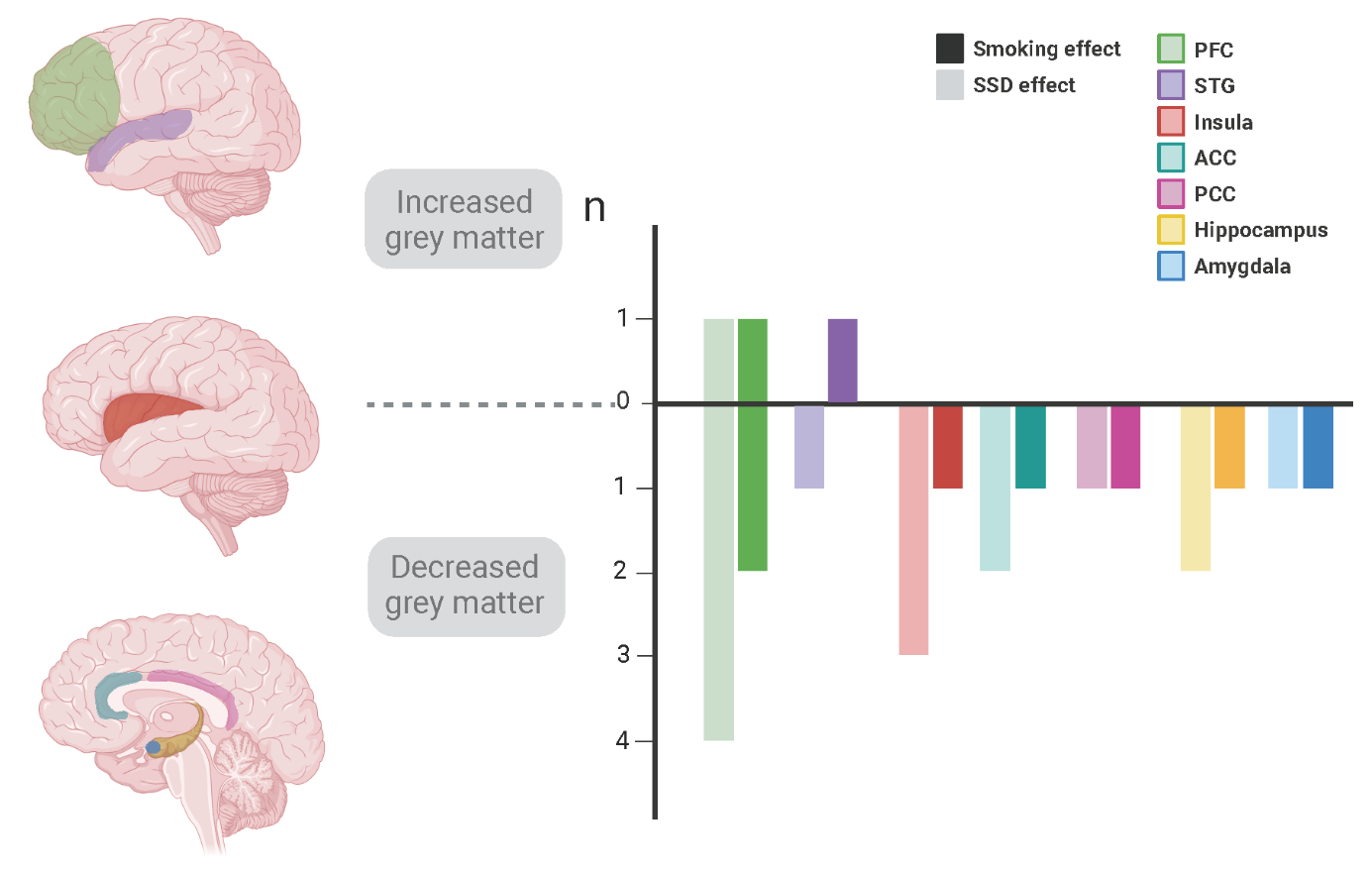
**

**S4 Regions with significant grey matter changes related to smoking and schizophrenia in structural MRI studies.** The count of papers with significant effects is displayed separately per region. Darker colours indicate schizophrenia effect and lighter colours indicate smoking effect. Specifically, prefrontal cortex reductions were observed specifically in the dorsolateral prefrontal cortex, medial orbitofrontal gyrus, medial frontal gyrus and inferior frontal gyrus bilaterally. Prefrontal cortex increases were demonstrated in the bilateral lateral orbitofrontal cortex and lateral prefrontal cortex. Figure created with BioRender.com. Abbreviations: ACC, anterior cingulate cortex; PCC, posterior cingulate cortex; PFC, prefrontal cortex; STG, superior temporal gyrus.

**S5 Outcomes of NIH quality assessment for all included studies.** Results are depicted per question of the quality assessment tool as well as the overall quality rating. Green denotes high quality (i.e. low risk of bias), orange denotes fair quality (i.e. fair risk of bias), and red denotes poor quality (i.e. high risk of bias). Grey denotes not applicable. Questions are listed below.

| **Article** | **Q1** | **Q2** | **Q3** | **Q4** | **Q5** | **Q6** | **Q7** | **Q8** | **Q9** | **Q10** | **Q11** | **Q12** | **Q13** | **Q14** | **Overall** |
| --- | --- | --- | --- | --- | --- | --- | --- | --- | --- | --- | --- | --- | --- | --- | --- |
| **DTI** | | | | |  |  |  |  |  |  |  |  |  |  |  |
| (Cullen et al., 2012) |  |  |  |  |  |  |  |  |  |  |  | NR |  |  |  |
| (Zhang et al., 2010) |  |  |  |  |  |  |  |  |  |  |  | NR |  |  |  |
| **Structural MRI** | | | |  |  |  |  |  |  |  |  |  |  |  |  |
| (Jørgensen et al., 2015) |  |  |  |  |  |  |  |  |  |  |  | NR |  |  |  |
| (Ringin et al., 2022) |  |  |  |  |  |  |  |  |  |  |  | NR |  |  |  |
| (Schneider et al., 2014) |  |  |  |  |  |  |  |  |  |  |  | NR |  |  |  |
| (Tregellas et al., 2007) |  |  |  |  |  |  |  |  |  |  |  | NR |  |  |  |
| (Van Haren et al., 2010) |  |  |  |  |  |  |  |  |  |  |  | NR |  |  |  |
| (Yokoyama et al., 2018) |  |  |  |  |  |  |  |  |  |  |  | NR |  |  |  |
| **Resting-state fMRI** | | | | |  |  |  |  |  |  |  |  |  |  |  |
| (Chen, 2023) |  |  |  |  |  |  |  |  |  |  |  | NR |  |  |  |
| (Fan et al., 2020) |  |  |  |  |  |  |  |  |  |  |  | NR |  |  |  |
| (Liao et al., 2019a) |  |  |  |  |  |  |  |  |  |  |  | NR |  |  |  |
| (Liao et al., 2019b) |  |  |  |  |  |  |  |  |  |  |  | NR |  |  |  |
| (Liu et al., 2018) |  |  |  |  |  |  |  |  |  |  |  | NR |  |  |  |
| (Moran et al., 2013) |  |  |  |  |  |  |  |  |  |  |  | NR |  |  |  |
| (Ward et al., 2022) |  |  |  |  |  |  |  |  |  |  |  | NR |  |  |  |
| (Yang et al., 2021) |  |  |  |  |  |  |  |  |  |  |  | NR |  |  |  |
| **Task-based fMRI** | | | | | |  |  |  |  |  |  |  |  |  |  |
| (Friedman et al., 2008) |  |  |  |  |  |  |  |  |  |  |  | NR |  |  |  |
| (Leyba et al., 2008) |  |  |  |  |  |  |  |  |  |  |  | NR |  |  |  |
| (Moran et al., 2018) |  |  |  |  |  |  |  |  |  |  |  | NR |  |  |  |
| (Potvin et al., 2016) |  |  |  |  |  |  |  |  |  |  |  | NR |  |  |  |
| (Potvin et al., 2017) |  |  |  |  |  |  |  |  |  |  |  | NR |  |  |  |
| (Potvin et al., 2019) |  |  |  |  |  |  |  |  |  |  |  | NR |  |  |  |

Abbreviations: NR, not reported; Q, question.

**Question 1:** Was the research question or objective clearly stated?

**Question 2:** Was the study population clearly specified and defined?

**Question 3:** Was the participation rate of eligible persons at least 50%?

**Question 4:** Were all the subjects selected or recruited from the same or similar populations (including the same time period)? Were inclusion and exclusion criteria for being in the study prespecified and applied uniformly to all participants?

**Question 5:** Was a sample size justification, power description, or variance and effect estimates provided?

**Question 6:** For the analyses in this paper, were the exposure(s) of interest measured prior to the outcome(s) being measured?

**Question 7:** Was the timeframe sufficient so that one could reasonably expect to see an association between exposure and outcome if it existed?

**Question 8:** For exposures that can vary in amount or level, did the study examine different levels of the exposure as related to the outcome (e.g., categories of exposure, or exposure measured as continuous variable)?

**Question 9:** Were the exposure measures (independent variables) clearly defined, valid, reliable, and implemented consistently across all study participants?

**Question 10:** Was the exposure(s) assessed more than once over time?

**Question 11:** Were the outcome measures (dependent variables) clearly defined, valid, reliable, and implemented consistently across all study participants?

**Question 12**: Were the outcome assessors blinded to the exposure status of participants?

**Question 13:** Was loss to follow-up after baseline 20% or less?

**Question 14:** Were key potential confounding variables measured and adjusted statistically for their impact on the relationship between exposure(s) and outcome(s)?

**References**

Chen, Y., 2023. Altered functional dynamics gradient in schizophrenia with cigarette smoking. Cerebral cortex (New York, N.Y. : 1991) 33(11), 7185–7192.

Cullen, K.R., Wallace, S., Magnotta, V.A., Bockholt, J., Ehrlich, S., Gollub, R.L., Manoach, D.S., Ho, B.C., Clark, V.P., Lauriello, J., Bustillo, J.R., Schulz, S.C., Andreasen, N.C., Calhoun, V.D., Lim, K.O., White, T., 2012. Cigarette smoking and white matter microstructure in schizophrenia. Psychiatry research 201(2), 152–158.

Fan, Y.S., Yang, S., Li, Z., Li, J., Guo, X., Han, S., Guo, J., Duan, X., Cui, Q., Du, L., Liao, W., Chen, H., 2020. A temporal chronnectomic framework: Cigarette smoking preserved the prefrontal dysfunction in schizophrenia. Progress in neuro-psychopharmacology & biological psychiatry 99, 109860.

Friedman, L., Turner, J.A., Stern, H., Mathalon, D.H., Trondsen, L.C., Potkin, S.G., 2008. Chronic smoking and the BOLD response to a visual activation task and a breath hold task in patients with schizophrenia and healthy controls. NeuroImage 40(3), 1181–1194.

Jørgensen, K.N., Skjærvø, I., Mørch-Johnsen, L., Haukvik, U.K., Lange, E.H., Melle, I., Andreassen, O.A., Agartz, I., 2015. Cigarette smoking is associated with thinner cingulate and insular cortices in patients with severe mental illness. Journal of psychiatry & neuroscience : JPN 40(4), 241–249.

Leyba, L., Mayer, A.R., Gollub, R.L., Andreasen, N.C., Clark, V.P., 2008. Smoking status as a potential confound in the BOLD response of patients with schizophrenia. Schizophrenia research 104(1-3), 79–84.

Liao, W., Ding, J., Marinazzo, D., Xu, Q., Wang, Z., Yuan, C., Zhang, Z., Lu, G., Chen, H., 2011. Small-world directed networks in the human brain: multivariate Granger causality analysis of resting-state fMRI. 2683–2694 54(4), 2683–2694.

Liao, W., Fan, Y.S., Yang, S., Li, J., Duan, X., Cui, Q., Chen, H., 2019a. Preservation Effect: Cigarette Smoking Acts on the Dynamic of Influences Among Unifying Neuropsychiatric Triple Networks in Schizophrenia. Schizophrenia bulletin 45(6), 1242–1250.

Liao, W., Yang, S., Li, J., Fan, Y.S., Duan, X., Cui, Q., Chen, H., 2019b. Nicotine in action: cigarette smoking modulated homotopic functional connectivity in schizophrenia. Brain imaging and behavior 13(6), 1612–1623.

Liu, H., Luo, Q., Du, W., Li, X., Zhang, Z., Yu, R., Chen, X., Meng, H., Du, L., 2018. Cigarette smoking and schizophrenia independently and reversibly altered intrinsic brain activity. Brain imaging and behavior 12(5), 1457–1465.

Moran, L.V., Betts, J.M., Ongur, D., Janes, A.C., 2018. Neural Responses to Smoking Cues in Schizophrenia. Schizophrenia bulletin 44(3), 525–534.

Moran, L.V., Sampath, H., Kochunov, P., Hong, L.E., 2013. Brain circuits that link schizophrenia to high risk of cigarette smoking. Schizophrenia bulletin 39(6), 1373–1381.

Potvin, S., Dugré, J.R., Fahim, C., Dumais, A., 2019. Increased Connectivity Between the Nucleus Accumbens and the Default Mode Network in Patients With Schizophrenia During Cigarette Cravings. Journal of dual diagnosis 15(1), 8–15.

Potvin, S., Lungu, O., Lipp, O., Lalonde, P., Zaharieva, V., Stip, E., Melun, J.P., Mendrek, A., 2016. Increased ventro-medial prefrontal activations in schizophrenia smokers during cigarette cravings. Schizophrenia research 173(1-2), 30–36.

Potvin, S., Tikàsz, A., Lungu, O., Stip, E., Zaharieva, V., Lalonde, P., Lipp, O., Mendrek, A., 2017. Impaired Coupling between the Dorsomedial Prefrontal Cortex and the Amygdala in Schizophrenia Smokers Viewing Anti-smoking Images. Frontiers in psychiatry 8, 109.

Ringin, E., Cropley, V., Zalesky, A., Bruggemann, J., Sundram, S., Weickert, C.S., Weickert, T.W., Bousman, C.A., Pantelis, C., Van Rheenen, T.E., 2022. The impact of smoking status on cognition and brain morphology in schizophrenia spectrum disorders. Psychological medicine 52(14), 3097–3115.

Schneider, C.E., White, T., Hass, J., Geisler, D., Wallace, S.R., Roessner, V., Holt, D.J., Calhoun, V.D., Gollub, R.L., Ehrlich, S., 2014. Smoking status as a potential confounder in the study of brain structure in schizophrenia. Journal of psychiatric research 50, 84–91.

Shafiei, G., Markello, R.D., Vos de Wael, R., Bernhardt, B.C., Fulcher, B.D., Misic, B., 2020. Topographic gradients of intrinsic dynamics across neocortex. eLife(9), e62116.

Tregellas, J.R., Shatti, S., Tanabe, J.L., Martin, L.F., Gibson, L., Wylie, K., Rojas, D.C., 2007. Gray matter volume differences and the effects of smoking on gray matter in schizophrenia. Schizophrenia research 97(1-3), 242–249.

Van Haren, N.E., Koolschijn, P.C., Cahn, W., Schnack, H.G., Hulshoff Pol, H.E., Kahn, R.S., 2010. Cigarette smoking and progressive brain volume loss in schizophrenia. European neuropsychopharmacology : the journal of the European College of Neuropsychopharmacology 20(7), 454–458.

Ward, H.B., Beermann, A., Nawaz, U., Halko, M.A., Janes, A.C., Moran, L.V., Brady Jr, R.O., 2022. Evidence for Schizophrenia-Specific Pathophysiology of Nicotine Dependence. Frontiers in psychiatry 13, 804055.

Yang, C., Tang, J., Liu, N., Yao, L., Xu, M., Sun, H., Tao, B., Gong, Q., Cao, H., Zhang, W., Lui, S., 2021. The Effects of Antipsychotic Treatment on the Brain of Patients With First-Episode Schizophrenia: A Selective Review of Longitudinal MRI Studies. Frontiers in psychiatry 12, 593703.

Yokoyama, N., Sasaki, H., Mori, Y., Ono, M., Tsurumi, K., Kawada, R., Matsumoto, Y., Yoshihara, Y., Sugihara, G., Miyata, J., Murai, T., Takahashi, H., 2018. Additive Effect of Cigarette Smoking on Gray Matter Abnormalities in Schizophrenia. Schizophrenia bulletin 44(3), 535–541.

Zhang, X., Stein, E.A., Hong, L.E., 2010. Smoking and schizophrenia independently and additively reduce white matter integrity between striatum and frontal cortex. Biological psychiatry 68(7), 674–677.
